# Video Remote Sign Language Interpreting and Health Communication for Deaf Patients: A Randomized Clinical Trial

**DOI:** 10.1001/jamanetworkopen.2025.57189

**Published:** 2026-02-04

**Authors:** Minerva Rivas Velarde, Laura Catalina Izquierdo Martinez, Jyoti Dalal, Angela Martinez-R, Karen Libey Guevara Rojas, Nicolas Alfonso Parra Valero, Danna Lesley Cruz Reyes, Jess Cuculick, Alexie Vallejo-Silva, Jonathan Irreño-Sotomonte, Nora Groce

**Affiliations:** 1 School Of Health Science, University of Applied Sciences and Arts Western, Geneva, Switzerland; 2 Institute of Ethics History and Humanities, Faculty of Medicine, University of Geneva, Geneva, Switzerland; 3 Institute of Global Health, Faculty of Medicine, University of Geneva, Geneva, Switzerland; 4 School of Medicine and Health Sciences, Universidad del Rosario, Bogotá, Colombia; 5 Departamento de Estadística, Facultad de Ciencias, Universidad Nacional de Colombia–Sede Bogotá, Bogotá, Colombia; 6 NTID Department of Liberal Studies, Rochester Institute of Technology, Rochester, New York; 7 Clínica de Nuestra Señora de la Paz, Bogotá, Colombia; 8 UCL International Disability Research Centre, Institute of Epidemiology and Healthcare, University College London, London, United Kingdom

## Abstract

**Importance:**

Current interpretation services for Deaf patients who use sign language are often ineffective or unacceptable. In-person interpretation is frequently unavailable, and while video remote interpreting (VRI) remains underused, its scalability may be a solution given interpreter shortages and cost barriers. Existing research focuses on user and interpreter preferences, leaving a critical gap in understanding how interpretation formats affect communication quality.

**Objective:**

To evaluate the effectiveness of VRI in improving communication outcomes between Deaf patients and physicians compared with usual communication tools, such as self-arranging interpretation, lip-reading, note-taking, and the use of images.

**Design, Setting, and Participants:**

This randomized clinical trial was conducted in Colombia at a public hospital from August 2023 to October 2024, involving Deaf adults who use Colombian Sign Language as their primary language. Participants were randomly assigned to either the control or intervention group. The data were analyzed between January and May 2025.

**Interventions:**

Patients were divided into 2 groups: an intervention group that received a medical appointment via VRI and a control group that received one via the current standard of communication. Both the Deaf participants and the health care professionals were blinded to the allocation.

**Main Outcomes and Measures:**

An assessment of communication using the Doctor-Patient Communication scale.

**Results:**

Data were collected from 210 Deaf participants, including 123 (58.6%) women and 87 (41.4%) men, with a mean (SD) age of 42 (13) years (range, 18-84 years). Overall, 108 participants (51.4%) reported using VRI. The intervention revealed that having VRI did not always result in improved communication between Deaf individuals and physicians. While those using VRI were more likely to report positive outcomes in certain areas, such as being encouraged to express themselves (odds ratio, 1.90; 95% CI, 1.13-3.18; *P* = .02), there was no difference in other areas, such as understanding the doctor (OR, 1.33; 95% CI, 0.79-2.23; *P* = .28).

**Conclusions and Relevance:**

In this randomized clinical trial of VRI in the health care context, some aspects of clinical communication were improved, but others were not. This suggests that critical preconditions have to be met for this technology to achieve its intended impact.

**Trial Registration:**

ClinicalTrials.gov Identifier: NCT05966623

## Key Points

**Question:**

Does the use of video remote interpreting (VRI) significantly improve communication outcomes compared with usual communication tools among Deaf patients?

**Findings:**

This randomized clinical trial involving 210 Deaf patients found that VRI did not always result in improved communication between Deaf individuals and physicians. While attentiveness, completeness, and other aspects of communication were rated more highly among participants using VRI, there were no differences in clarity and feeling listened to, among others.

**Meaning:**

The findings of this randomized clinical trial suggest that VRI alone is not sufficient to ensure quality communication between health care professionals and Deaf individuals.

## Teaser

This randomized clinical trial evaluates whether the use of video remote interpreting among Deaf patients in Colombia improves the quality of their communication with health care professionals.

## Introduction

Despite the widespread adoption of the Convention on the Rights of Persons with Disabilities, which advocates for equitable health services for persons with disabilities, including Deaf persons, access to quality professional sign language interpretation remains a global issue.^1^ In Colombia, the law stipulates that interpreters for Deaf individuals must be provided in health care and other public services^2^; however, the implementation of this law is severely limited. Many Deaf individuals continue to face challenges in accessing the health care system. While some rely on in-person interpreters for effective communication with health care practitioners, they still encounter difficulties due to geographic, time, and financial constraints related to booking sign language interpreters.^3^

In Colombia, no institutional or governmental policy promotes comprehensive training and qualification in sign language interpreting.^4^ Currently, most interpreters are trained by different Deaf person associations or local or national nongovernmental organizations. Despite the creation of professional training programs, most of which are privately offered, these programs operate in parallel. Interpreters are not obligated to provide evidence of training or certification through examinations to validate their competencies. Certificates issued by nongovernmental organizations or Deaf associations simply indicate a level of sign language proficiency. At present, there are no clear guidelines or regulations defining what agencies or groups should do to guarantee the quality of interpreters’ education and their skill level when providing interpreting services.

In response to the communication needs of the Deaf population, the growing adoption of video remote interpreting (VRI) can be seen as a digital solution that enables Deaf individuals to communicate effectively with the health care system. However, in practice, health care professionals, Deaf individuals, and sign language interpreters often highlight challenges that hinder its effective use in certain health care situations, such as technical issues and logistical barriers.^3^

There is no reliable data on the availability of VRI or in-person interpretation.^3^ While in-person qualified sign language interpretation in the health care setting is considered the ideal service provision standard, this is mainly unavailable even in high-income countries due, among others, to the severe shortage of interpreters.^5^ Given limited human resources and their associated costs, VRI is often seen as a scalable and sustainable alternative to in-person interpretation.

Prior evidence from spoken language suggests that patients have strong preferences for in-person interpretation over remote services due to improved rapport-building, trust, and perceived communication quality.^3,6,7,8^ However, this remains unknown for Deaf persons, who communicate primarily using sign language.^3^ A study conducted in the United States surveyed 968 Deaf adults regarding their use of VRI in health care. The results indicated that 413 of these individuals had not used VRI within the past 12 months.^3^ Additionally, another study in Colombia, which also involved a survey of Deaf individuals, revealed that only 42% of the 204 participants reported using VRI services in health care settings.^9^ Poor health outcomes observed among Deaf individuals, such as higher risks of cardiovascular disease, diabetes, depression, and obesity, underscore the importance of evaluating the effectiveness of VRI in overcoming barriers to quality communication in health care settings.^10^

Key determinants for the use of VRI for sign language include internet access, device availability, and keeping devices charged, a common systems issue. Additionally, 80% of Deaf persons live in low- and middle-income countries (LMICs) and are overrepresented among those living in poverty.^11^ There is limited evidence on digital determinants of health and overall health care access for Deaf persons, particularly in LMICs. Dalal et al^9^ showed that in Colombia, mobile phone access reached approximately 70% of Deaf persons. Thus, Deaf persons use the internet 28% less than hearing people.^12^ Regarding sign language interpretation, results show that less than half of Deaf persons regularly access VRI or any form of digital tool for communication during medical consultations.^9^

The question guiding this study is: does the use of VRI significantly improve communication outcomes compared with usual communication tools among Deaf patients? This article focuses on persons whose primary language is sign language. In international literature, *Deaf* with capital D is used when the person identifies as a member of the Deaf community and their language is sign language.^13^

## Methods

This randomized clinical trial (RCT) is part of a larger study that included a national survey identifying Deaf health care priorities^9,14^ and a qualitative exploration of the intervention’s social context.^5^ The RCT tested an intervention informed by these findings. The study was overseen by a steering committee that included local and international Deaf organizations and global health experts throughout its development.

### Design

This RCT was conducted at Clinica de Nuestra Señora de la Paz in Bogotá, Colombia, from August 2023 to October 2024. Participants were randomly assigned to either the intervention group, which received a medical appointment using VRI, or the control group, which received a medical appointment using standard of care available, namely, communication methods such as formal or informal interpretation, lip-reading, note-taking, or mobile phones. The research protocol was authorized by the ethics committee of the University of Geneva on research involving humans, the ethics committee of Clinica de Nuestra Señora de la Paz, and the ethics committee of Universidad del Rosario

Patient outcomes were assessed using the Doctor-Patient Communication (DPC) scale,^15^ administered after the consultation. The informed consent form was explained to all participants with the assistance of a Colombian Sign Language (CSL) interpreter, and participants gave their written permission. All participants received COP 20 000 (US $4.50) for their participation. The project also covered transportation costs. The study protocol has been published previously^16^ and is available in Supplement 1. This study adhered to the Consolidated Standards of Reporting Trials (CONSORT) reporting guideline.^17^ For the interpretation during the intervention, we collaborated with experienced CSL interpreters, who held the highest level of accreditation available at the time, to facilitate communication between health care professionals and Deaf participants during consultations.

### Participants

**Figure 1.**
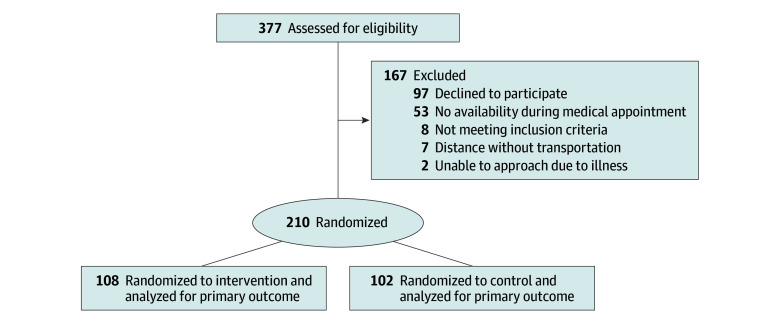
Study Flow Diagram

Eligible participants were required to be 18 years or older; reside in Bogotá, Colombia; and be fluent in CSL. In addition, they must possess sufficient sensorimotor, cognitive, and communication skills to interact independently with health care personnel. Participants were excluded if they had additional impairments that affected language development or the use of sign language. See Figure 1 for the CONSORT flow diagram.

### Sample Size

To test whether the group that used VRI had better communication outcomes than the control group, we computed the sample sizes needed.^16^ For each group, we needed 97 participants. There were no dropouts. We included data from 108 people using VRI and 102 from the group not using VRI, amounting to a total of 210 participants.

### Randomization

To prevent selection bias, participants were randomly assigned to intervention or control groups using block randomization. A block size of 4 (2 per group) was used to ensure balanced allocation. Randomization lists were generated with a web-based application,^18^ following predefined procedures. Each participant was randomly assigned within blocks (eg, ABBA, BAAB) until all participants had been allocated. To maintain allocation concealment, block sizes were undisclosed. At the medical appointment, participants drew sealed envelopes containing their group assignment and participant code. Group assignment was revealed only upon opening the envelope, ensuring blinding for the participant, researcher, general practitioner, and sign language interpreter until the point of care.

### Recruitment

The participants were able to learn about the study through social media, advocacy organizations, and local networks. A researcher, along with a sign language interpreter, contacted each potential participant through a WhatsApp video call—the key communication channel among Deaf individuals in Colombia using sign language—to explain the project and schedule a medical appointment. During recruitment, a research assistant conducted a brief interview with each potential participant to assess their ability to communicate in sign language and to make independent decisions regarding their health.

### Interventions

All participants were seen on an ambulatory or outpatient basis at the hospital. Upon arrival at the hospital, participants were greeted by a research assistant who facilitated their admission process. They were then guided to the consultation room, where they engaged in a standard medical consultation known in the Colombian health system as “standard promotion and disease prevention or annual check.” These consultations are preventive and nonurgent in nature. During consultations, the health care professional conducted a medical history assessment, performed a physical examination, and provided education and recommendations based on the findings. For participants in the intervention group, VRI was used during the appointment. A 32-inch screen displayed the CSL interpreter, who connected through the Zoom (Zoom Video Communications) platform. The participants in the control group had access to the same intervention, albeit without VRI contact. They communicated with the physician using standard communication methods (eg, self-arranging interpretation, lip-reading, note-taking, or the use of images).

After the medical consultation, participants completed the DPC scale. This scale was administered by a research assistant using a tablet and was available on the LimeSurvey platform with Spanish subtitles and CSL videos.

### Outcomes

The primary outcome measure was an assessment of communication outcomes using the DPC (Table 1). The DPC consists of 13 questions.^15^ Each question had 4 response options: no, possibly no, possibly yes, and yes. The DPC scale has high internal consistency (Cronbach α = 0.89) and good external validity. It is a user-friendly and validated generic questionnaire designed to assess communication in the context of acute conditions, suitable for both clinical research and routine practice. It measures (1) the creation of a good interpersonal relationship, (2) the exchange of information, and (3) the making of treatment-related decisions that involved patients in decision-making.

**Table 1.** Doctor-Patient Communication (DPC) Scale^a^

Questions	Score by answer, points			
	No	Possibly no	Possibly yes	Yes
Q1, listening: did the doctor listen to you carefully during the consultation?	1	2	3	4
Q2, patience: did the doctor allow you to talk without interrupting you?	1	2	3	4
Q3, attentiveness: did the doctor encourage you to express yourself/talk?	1	2	3	4
Q4, physical examination: did the doctor examine you thoroughly?	1	2	3	4
Q5, empathy: do you feel that the doctor understood you?	1	2	3	4
Q6, clarity: was it easy to understand what the doctor said?	1	2	3	4
Q7, completeness: do you feel you were given all the necessary information?	1	2	3	4
Q8, disadvantages: did the doctor explain the advantages and disadvantages of the treatment or care strategy?	1	2	3	4
Q9, decisional involvement: did the doctor involve you in the decision-making?	1	2	3	4
Q10, reassurance: in your opinion, did the doctor have a reassuring attitude and way of talking?	1	2	3	4
Q11, understanding: did the doctor make sure that you understood his explanations and instructions?	1	2	3	4
Q12, confidence: do you have confidence in this doctor?	1	2	3	4
Q13, concerns: did the doctor reply to all your expectations and concerns?	1	2	3	4

Abbreviation: Q, question.

^a^ The Doctor-Patient Communication scale was developed by Sustersic et al,^15^ 2018.

This scale was translated into CSL following a rigorous cultural adaptation and translation procedure by Deaf individuals. The translation team consisted of 1 hearing and 2 Deaf individuals, all bilingual in Spanish and CSL. They were Colombian, professionally accredited translators and interpreters residing in Colombia, with prior experience in survey translation. The team jointly discussed and agreed on the appropriate translation of each question before performing the forward translation from Spanish into CSL. For the video recordings, a Deaf signer who used CSL as their preferred language appeared on screen. They stood upright in the center, with their upper body and hands clearly visible. To minimize visual distractions, no jewelry was worn and a plain, unpatterned background was used. The video dimensions were adjusted to ensure optimal viewing on a tablet.^14^ The videos were hosted on the LimeSurvey platform, presented in CSL, written in Spanish, and played on a tablet. Participants completed the DPC scale after leaving the medical consultation.^15^

### Statistical Analysis

The DPC scale consisted of 13 questions (Q1-Q13) adapted from Sustersic et al^15^ (Table 1), assessing various aspects of the consultation, including clarity, completeness of information, and involvement in decision-making, among others.^15^ Each of these questions had 4 ordered response categories: no, possibly no, possibly yes, and yes, reflecting an increment in the levels of positive experience or agreement as we go from no to yes. As the responses were ordered but not equally spaced, we analyzed them as ordinal variables.

To compare the distribution of ordinal responses across the 13 items between 2 groups (ie, people using VRI and people not using VRI), we used the nonparametric Mann-Whitney *U* test,^19^ a robust method for assessing differences in ordinal or nonnormally distributed data. For each item, we report the Mann-Whitney *U* statistic, associated *P* value, and group medians, along with the direction of differences (VRI better than no VRI, VRI worse than no VRI, or equal).

To quantify the direction and magnitude of associations while accounting for the ordinal nature of the responses, we fitted ordinal logistic regression (OLR) models for each of the 13 items. We coded the outcome variables as ordinal with 4 ordered levels: no, 0; possibly no, 1; possibly yes, 2; and yes, 3, reflecting increasing agreement. This coding preserves the natural ranking of responses and is appropriate for ordinal logistic regression. VRI usage (1, using VRI; 0, not using VRI) was included as the independent variable. For each OLR model, we report odds ratios (ORs), 95% CIs, and the corresponding *P* values.

We assessed the model fit using the Akaike information criterion (AIC) and log-likelihood. We evaluated the proportional odds assumption by comparing the full ordinal logistic model with separate binary logistic regressions at each threshold. We also used an approximate likelihood ratio test (LRT) to compare nested models. We report AIC, log-likelihood, LRT statistic, and corresponding *P* values to assess model fit (eTable in Supplement 2).

To assess whether randomization resulted in balanced groups, we ran a logistic regression with VRI usage as the outcome and participant’s education level, age, and sex as the independent variable. We also report pairwise correlations among education status, age, and sex to explore potential collinearity among these covariates:

VRI usage = education status + age + sex,

where VRI usage indicates using VRI or not, and education status ranged from 1 to 7, with higher values indicating greater years of education.

All statistical tests were 2-tailed, and *P* values less than .05 were considered statistically significant. We conducted all analyses in Python version 3.7.4 (Python Software Foundation) using pandas, numpy, scipy, and statsmodels packages.

## Results

We included data from 210 Deaf participants, 123 (58.6%) women and 87 (41.4%) men. A total of 108 participants (51.4%) reported using VRI. The age of the participants ranged from 18 to 84, with a mean (SD) age of 42 (13) years and a median (IQR) of 40 (32-50) years. Overall, 65 people (31.0%) reported having upper secondary education (10th-11th grade), and 29 (13.8%) participants had university education (Table 2).

**Table 2.** Participant Characteristics

Variables	Participants, No. (%) (N = 210)
Age, y	
Mean (SD)	42 (13.1)
Median (IQR)	40 (32-50)
Sex	
Men	87 (41.4)
Women	123 (58.6)
VRI usage	
Using VRI	108 (51.4)
Not using VRI	102 (48.6)
Education	
Upper secondary education (10th-11th grade)	65 (31.0)
Technical or technological education	52 (24.8)
Primary education (1st-5th grade)	37 (17.6)
University education	29 (13.8)
Lower secondary education (6th-9th grade)	17 (8.1)
No formal education	7 (3.3)
Postgraduate education	3 (1.4)

Abbreviation: VRI, video remote interpreting.

Significant differences were observed for 4 items on the PDC scale: attentiveness (Q3; OR, 1.90; 95% CI, 1.13-3.18; *P* = .02), physical examination (Q4; OR, 2.19; 95% CI, 1.17-4.08; *P* = .01), completeness (Q7; OR, 1.90; 95% CI, 1.12-3.21; *P* = .02), and decisional involvement (Q9; OR, 2.49; 95% CI, 1.48-4.19; *P* < .001) (Table 3). Both the ORL and the Mann-Whitney *U* test showed higher ratings among participants using VRI for these items, suggesting that for these domains, participants using VRI perceived more effective communication compared with those not using VRI.

**Table 3.** Results of OLR and MWU Tests for All 13 Questions

Question	OLR		MWU	
	OR (95% CI)	*P* value	Direction of median scores	*P* value
***P* < .05**				
Q3: attentiveness	1.90 (1.13-3.18)	.02	VRI better than no VRI	.02
Q4: physical examination	2.19 (1.17-4.08)	.01	VRI better than no VRI	.01
Q7: completeness	1.90 (1.12-3.21)	.02	VRI better than no VRI	.02
Q9: decisional involvement	2.49 (1.48-4.19)	<.001	VRI better than no VRI	<.001
***P* < .10**				
Q5: empathy	1.60 (0.94-2.72)	.08	VRI better than no VRI	.08
Q8: disadvantages	1.69 (0.97-2.94)	.06	Equal	.06
Q12: confidence	1.65 (0.98-2.77)	.06	VRI better than no VRI	.06
***P* > .10**				
Q1: listening	1.30 (0.75-2.25)	.34	Equal	.34
Q2: patience	1.35 (0.79-2.30)	.27	Equal	.27
Q6: clarity	1.46 (0.86-2.47)	.16	VRI better than no VRI	.16
Q10: reassurance	1.43 (0.81-2.55)	.22	Equal	.22
Q11: understanding	1.33 (0.79-2.23)	.28	VRI better than no VRI	.28
Q13: concerns	1.30 (0.75-2.25)	.34	Equal	.34

Abbreviations: MWU, Mann-Whitney *U*; OLR, ordinal logic regression; OR, odds ratio; Q, question.

No differences were found for empathy (Q5; OR, 1.60; 95% CI, 0.94-2.72; *P* = .08), disadvantages (Q8; OR, 1.69; 95% CI, 0.97-2.94; *P* = .06), and confidence (Q12; OR, 1.65; 95% CI, 0.98-2.77; *P* = .06). Although these results were not statistically significant, the direction of effects (ORs >1.50, favoring VRI over no VRI) suggests potential improvements in perceived communication among VRI users for these items. For the remaining 6 items—listening (Q1), patience (Q2), clarity (Q6), reassurance (Q10), understanding (Q11), and concerns (Q13)—both tests indicated no statistically significant differences between the two groups (all *P* > .10).

All the LRT *P* values were greater than .99, indicating no violation of the proportional odds assumption and confirming that the ordinal logistic models were appropriate for all 13 questions. For 4 questions, the Mann-Whiney *U* statistics and median scores showed statistically significant differences between the 2 groups (Q3, attentiveness; Q4, physical examination; Q7, completeness; and Q9, decisional involvement), with median scores higher among VRI users, supporting the main findings. However, median responses were identical for the other questions (eTable 1 in Supplement 2).

### Association of Demographic Characteristics With VRI Usage

#### Pairwise Correlations

**Figure 2.**
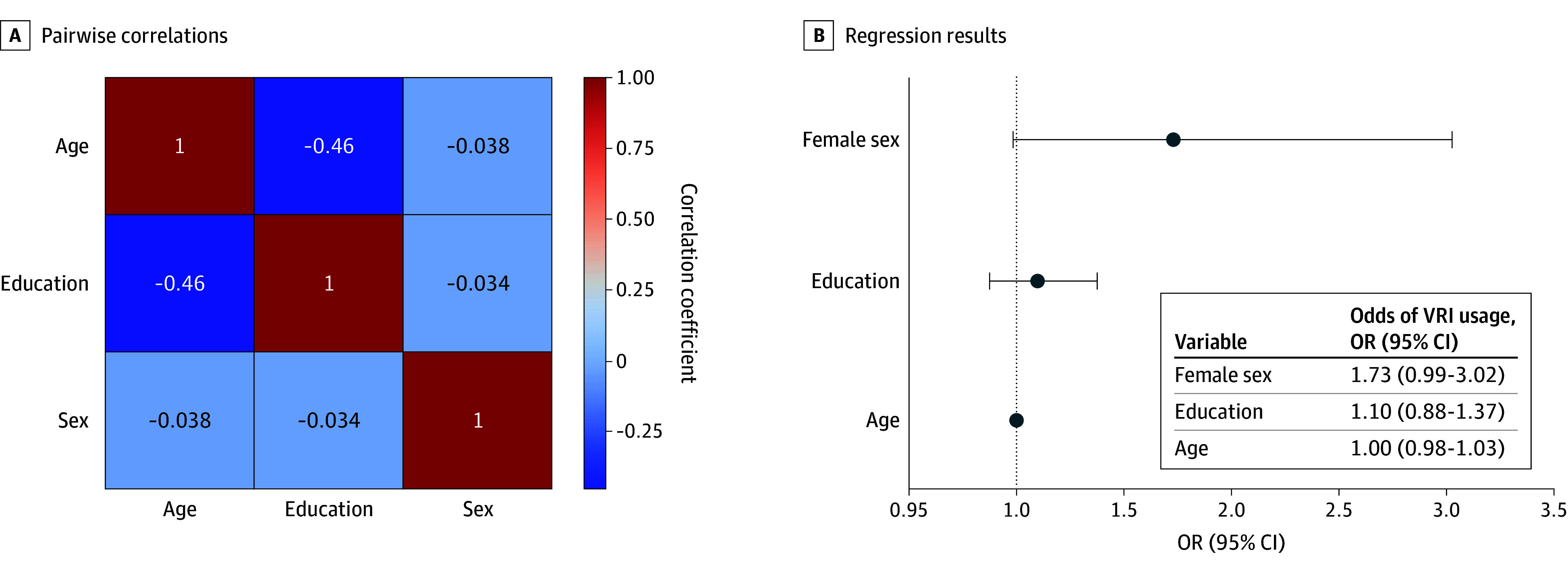
Correlations Between Demographic Characteristics and Association Between Demographic Characteristics and Video Remote Interpreting (VRI) Usage OR indicates odds ratio.

We found a moderate negative correlation (correlation coefficient = −0.46) between education and age (Figure 2A). Older individuals generally had lower levels of education, whereas younger individuals tended to have completed more years of schooling. Other pairwise correlations were nearly 0 or negligible.

#### Regression Results

As expected, none of the independent variables significantly explained VRI usage: sex (OR, 1.73; 95% CI, 0.99-3.02), education status (OR, 1.10; 95% CI, 0.88-1.37), and age (OR, 1.00; 95% CI, 0.98-1.03). This suggests that the randomization process was successful and groups were balanced across key demographics (Figure 2B).

## Discussion

The intervention revealed that using VRI did not always result in improved communication between Deaf individuals and physicians. While those using VRI were more likely to report positive outcomes in certain areas, such as being encouraged to express themselves, receiving thorough examinations, being given information, and being involved in decision-making, it made no significant difference in other areas. These included understanding the doctor, speaking without interruptions, perceiving the doctor as having a reassuring attitude, or feeling assured that the doctor understood them. Additionally, VRI users did not report improvements in feeling that the doctor responded to all their concerns, feeling understood, feeling able to understand the advantages and disadvantages of treatment, or having confidence in the doctor.

To our knowledge, this is the first study anywhere, in the global North or South, to examine the efficacy of the VRI in communication outcomes among Deaf patients. While our study did not show consistent communication benefits to VRI use across all domains measured, it was beneficial for the domains of being encouraged to express themselves, receiving thorough examinations, being given information, and being involved in decision-making. Although it is interesting that the technology had a positive impact on some aspects of communication in the health care context, these improvements are not sufficient to judge the intervention favorably.

Limited health literacy and mistrust toward interpretation personnel may have affected participants’ engagement with VRI and their perceptions of communication quality. Our prior work highlighted that low literacy levels, particularly health literacy, among Deaf individuals were a significant concern.^7^ This issue is well-documented in global health literature, where low literacy rates within the Deaf population are recognized as a persistent challenge.^16^ We also documented a prevailing mistrust toward interpretation personnel, which appears to stem from weak training and governance structures, as well as ongoing political tensions between disabled persons’ organizations and sign language interpreters’ unions.^5^ This study highlights the broader digital and social determinants of health affecting the Deaf population in Colombia, an LMIC, which may also resonate with Deaf populations across the global South.

VRI services are prone to more technical and logistical barriers due to the lack of familiarity regarding their use by health care professionals and Deaf users.^3^ However, the results of this study showed that even if all technical and economic barriers were removed, issues remain. Our study suggests that to effectively utilize VRI, several preconditions should be met. These could include strong governance mechanisms to ensure accountability, trust between users and physicians, and adequate health literacy among users. Without meeting these preconditions, new technologies could expose persons with disabilities to new forms of vulnerability.

This finding aligns with evidence that raises concerns about how health governance is or is not efficiently protecting vulnerable populations from harm stemming from new technologies.^20,21^ Similarly, in many LMICs, the implementation of VRI technology, designed to alleviate communication barriers for Deaf individuals in health care, might inadvertently exacerbate social segregation. The lack of access could become obscured by ineffective or incomplete solutions.

### Limitations

This study has limitations. We acknowledge that self-reported data may be subject to bias or imprecision. The DPC scale used in this study was centered according to the patient perspective, which is why we include interviews with health care professionals and sign language interpreters in other phases of the project. Additionally, given the nature of the intervention, blinding was not possible; however, efforts were made to minimize bias. The researcher, participant, general practitioner, and sign language interpreter only became aware of the participant’s group allocation when the sealed envelope was opened at the start of the medical consultation.

## Conclusions

In this RCT of VRI in the health care context, use of VRI improved some aspects of patient-physician communication, including feeling that the physician was attentive, that the appointment was complete, and that the patient was involved in decision-making. However, there were no differences in other aspects, including listening, patience, clarity, and understanding, among others. The study provides robust evidence regarding how digital and social factors affect the outcomes of e-health interventions for access to health care for the Deaf population. The findings suggest that some preconditions—including literacy levels among Deaf individuals; training, oversight, and quality assurance mechanisms for interpreters, and addressing infrastructural barriers, such as access to devices and reliable internet connectivity—must be met for VRI technology to achieve its intended impact. E-health is a rapidly growing area in health care. VRI helped to enhance services, improve surveillance, and enable research; however, our findings suggest that while some barriers to health care could be solved with technology, other barriers could be exacerbated.

## Notes

Supplement 1.
Trial Protocol

Supplement 2. **eTable.** Ordinal Logistic Regression Fit Statistics and Mann-Whitney *U* Test (*U* Statistic and Median Scores) Results for 13 Communication Questions Comparing VRI Users and Nonusers

Supplement 3.
Data Sharing Statement
