# Error in Affiliation

**DOI:** 10.1001/jamanetworkopen.2026.5103

**Published:** 2026-03-10

In the Original Investigation titled “Video Remote Sign Language Interpreting and Health Communication for Deaf Patients: A Randomized Clinical Trial,”^1^ published February 4, 2026, there was an error in Danna Lesley Cruz Reyes’s affiliation. It should have read Departamento de Estadística, Facultad de Ciencias, Universidad Nacional de Colombia–Sede Bogotá, Bogotá, Colombia. This article has been corrected.^1^
