# Supplementary material for: Video Remote Sign Language Interpreting and Health Communication for Deaf Patients: A Randomized Clinical Trial
Source: JAMA Netw Open. 2026 Feb 4;9(2):e2557189. doi: 10.1001/jamanetworkopen.2025.57189 (PMC12873765; doi:10.1001/jamanetworkopen.2025.57189)
Supplement: Supplement 1. — Trial Protocol [file jamanetwopen-e2557189-s001.pdf]

Clinical Study Protocol

Assessing the Impact of Video Remote Sign Language Interpreting in Healthcare: Linking Disability Studies with Empirical Challenges of Public Health Research

This is a Randomise study that looks at what is the effectiveness of the VRI system in improving communication outcomes between Deaf patients and doctors versus the ‘available standard of care of the usual communication tools, including informal interpretation, lip or note reading, using their mobile phones to contact a formal or informal interpreter, for Deaf patients aged 18 and older in Bogota Colombia

Study Type:	*Randomise Control Trial*
Study Categorisation:	*Minimal risk*
Sponsor, Sponsor-Investigator or Principal Investigator:	*Swiss Nacional Science Fundation,*<br>*RIVAS VELARDE Minerva*<br>*iEH2 - Institute for Ethics, History, and the Humanities*<br>*Centre médical universitaire*<br>*Mobile +41774562383*<br>*1 rue Michel Servet*<br>*CH - 1211 Geneva 4*

Background and Rationale:

In-person or VRI sign language interpretation is largely unavailable. In a scoping review, we identified a knowledge gap regarding the quality of interpretation and training in sign language interpretation for health care. We also found that this area is under-researched, and the evidence is scant. All available evidence came from high-income countries, which is particularly problematic given that most DHH persons live in low- and middle-income countries. Thus, the available literature shows that VRI may enable deaf users to overcome interpretation barriers and can potentially improve communication outcomes between them and health personnel within health care services. For VRI to be acceptable, sign language users require a VRI system supported by devices with large screens and a reliable internet connection, as well as qualified interpreters trained in medical interpretation There is no clear data on the availability of VRI or in-person interpretation. Given the cost, VRI may be more available than in person. Available data tend to focus on assessing personal references of Deaf users in regards to interpretation, as well as interpreters' preferences and maximising recourses allocation.

Objective(S):

To assess the effectiveness of the VRI system in improving communication outcomes between Deaf patients and doctors

Produce a VRI model addressing the challenges faced by Deaf people that will be tested, implemented, and sustained in Bogota Colombia.

Outcome(s):

Score in an easy-to-use and validated generic questionnaire to assess DPC in the context of acute conditions, usable both in clinical research and in routine practice that measures

A: creating a good interpersonal relationship,

B: exchanging information, and

C: Making treatment-related decisions that involve the patients in decision-making

Secondary outcomes

A: Health profile of Deaf persons to be compared against non-deaf persons

Study design:

Randomised, As the comparator treatment will be usual care.6-month trial

Arm	Intervention
Experimental VRI sign language interpretation<br>Participants will be welcome from their entry point to the hospital, they will attend a general check-up with a GP or other services and be provided with VRI until they leave	Participants will be provided with a Tablet of 14' inches with interrupted VRI in Colombian Sign Language. Professionally accredited Sign language interpreter. At the end of the hospital visit, they will complete a scale measuring Doctor-Patient-Communication
Experimental: primo control<br>Participants get welcome at the entry point of the hospital, they will attend a general check-up with a GP or other services and	Participants get welcome at the entry point of the hospital, they are not provided with VRI. At the end of the hospital visit, they will complete a scale measuring Doctor-Patient-

they are not provided with by the study with VRI	communication

Explanation for choice of comparator

In-person or VRI sign language interpretation is largely unavailable. Thus, there is no clear data on the magnitude of the availability gap of VRI or in-person interpretation. In-person qualified sign language interpretation in the healthcare setting tends to be described as the ideal standard of service provision. Thus, it is largely unavailable even in HIC. The assumption is based upon minimal available evidence on the personal preferences of Deaf persons in the USA. There is no evidence that in-person interpretation is efficient in the context of weaker infrastructure such as low sign language literacy rates across Deaf persons, lack of standard qualification of interpreters and lack of interpreters and sustainable financing in HIC. To my knowledge, there is no study assessing DPC while using sign language interpretation.

Given the cost, VRI may be more sustainable than in-person. Assessing the efficiency of VRI versus the standard of care would be d of more value given that there is no other effective intervention to compare.

Population and sampling

The sample size for this trial is based upon a registry of persons who seek care at partner hospitals and registered Deafness on their health records. Deaf persons tend to be quite disengaged from the health system as stated above, this is a hard-to-rich population. The of 330-patient registry Deaf patients is highly unique and rather large given that the prevalence of deafness is from 0.2 to 0.9 in LMIC and lower in HIC. A definitive RCT will be deemed feasible when at least 120 individuals accept partaking in the study.

This sample generates reliable SE, SD and 95% CI on the sample size required for the large RCT with this measure as the primary outcome.

Method of assignment

A random allocation list will be created at randomization.com. This site would allow randomizing in blocks 4, 6 and 8. Including 20 blocks of each type will produce a random list of 330. Groups stratifying on gender and age (below 73 years/73+ years) to ensure even distribution of these variables. The block sizes will not be disclosed to ensure concealment.

The online output can be transferred to Word and edited, and the numbered allocations (from 1 to 330) are put in sealed envelopes, with the envelopes numbered.

When calling potential participants, they will be asked to participate if they agree, the envelope will be opened, and the participants will be assigned to a group. Blank spots will be listed to allocate patients outside the initial list that may arrive at the hospital during the trial.

All this paperwork will be filed for quality assurance purposes.

Sequence

A researcher will contact the Deaf patient by VRI.

The trial coordinator will ask, do you communicate using Colombian Sign Language (in CSL) the answer is yes. The coordinator shall ask if this is his/hers preferred language.

They will judge if the patient communicates using sign language and hold enough sensorimotor, cognitive and communication skills to communicate independently with health personnel.

If yes, the study will be explained, and the patient will be invited to a general medical check-up. If they agree to participate, the researcher will open the envelope with their number. Then the researcher will learn where to assign the patient to the control group of the intervention.

The Control group will be assigned a date on which no VRI interpreter will be available.

The intervention group will be assigned a date on which VRI will be available.

Single-blinded –

The researcher needs to learn before the patient comes to the hospital as VRI needs to be organised. Thus, they will only know if the participants shall be assigned to the intervention or control group after the patient agrees to take part. Double blinding is not feasible as interpreters will not be available 24x7.

Inclusion / Exclusion criteria:

Age: 18 +

Sex: all

Accepts healthy volunteers: Yes

Inclusion Criteria

- Uses Colombian Sign Language as the preferred language.
- Enough sensorimotor, cognitive and communication skills to communicate independently with health personnel

Exclusion criteria

- Does not communicate using sign language.
- Additional impairments which affect language development or the use of sign language.
- Refusal of the participant's representative(s) to participate in the study,
- Refusal of the participant's representative(s) to participate in a modality of the study,

Measurements and procedures:

The participants assigned to the intervention group will receive a general medical check-up

This shall include a general practitioner or a nurse– who will ask you about:

Lifestyle and family history,

Measure height and weight,

Take blood pressure

A communication scale will be applied directly after the consultation. The selected' scale assesses

A: creating a good interpersonal relationship,

B: exchanging information, and

C: Making treatment-related decisions that involve the patients in decision-making

This scale has been already linguistically and culturally adapted to Colombian Sign Language

Communication outcomes using the following tool

QUESTIONS	No	Possi—bly no	Possi-bly yes	Yes
1. Did the doctor listen to you carefully during the consultation?	1	2	3	4
2. Did the doctor allow you to talk without interrupting you?	1	2	3	4
3. Did the doctor encourage you to express yourself / talk?	1	2	3	4
4. Did the doctor examine you thoroughly?	1	2	3	4
5. Do you feel that the doctor understood you?	1	2	3	4
6. Was it easy to understand what the doctor said?	1	2	3	4
7. Do you feel you were given all the necessary information?	1	2	3	4
8. Did the doctor explain the advantages and disadvantages of the treatment or care strategy?	1	2	3	4
9. Did the doctor involve you in the decision-making?	1	2	3	4
10. In your opinion, did the doctor have a reassuring attitude and way of talking?	1	2	3	4
11. ***Do you think the doctor was in general respectful?****	1	2	3	4
12. Did the doctor make sure that you understood his explanations and instructions?	1	2	3	4
13. ***Do you think the doctor told the whole truth?****	1	2	3	4
14. Do you have confidence in this doctor?	1	2	3	4
15. Did the doctor reply to all your expectations and concerns?	1	2	3	4

Study Product / Intervention:

Participants will be provided with a Tablet of 17’ inches with interrupted VRI in Colombian Sign Language. Professionally accredited Sign language interpreter. With training on health interpretation. At the end of the hospital visit, they will complete a scale measuring Doctor-Patient-Communication

Control Intervention (if applicable):

No VRI will be assigned, participants are free to use any interpretation tool and support as they would normally do if any.

The participants assigned to the intervention group will receive a general medical check-up

This shall include a general practitioner or a nurse– who will ask you about:

Lifestyle and family history,

Measure your height and weight,

Take your blood pressure

A communication scale will be applied directly after the consultation.

Number of Participants with Rationale:

For 2 x 150-200 respondents. The outcome variable is a communication score; a sample size of 2x84 will detect a difference between means of 0.5 standard deviations (with a power of 0.90), and 2x340 would detect a difference of 0.25 SD. Even if the intervention is very effective, the instrument may lack reliability, which would dilute the contrast.

inclusion of a non-deaf sample from the same consultations would be useful, to provide a reference point against which the (possible) success of the intervention can be assessed.

Study Duration:

12 months

Investigator(s):

Dr. Minerva Rivas Velarde (Team leader, UNIGE)

Laura Izquierdo Martinez, Research assistant, Faculty of Medicine, Del Rosario University

Prof. Angela Martinez Rodriguez, Prof. Speech pathology and linguist, Faculty of Medicine, Del Rosario University/Mederi Hospital

Prof. Danna Lesley Cruz Reyes, Prof. Statistics and Mathematics, Faculty of Medicine, Del Rosario University

Study Centre(s):

Faculty of Medicine, Del Rosario University/Mederi Hospital

Study schedule

Study Periods	*2 month*	*8 months*	*2 months assessments*
Enrolment			
Generation of a random allocation list	*x*		
Call from the researcher	*x*		
In- /Exclusion Criteria	*x*		
Patient Information and Informed Consent	*x*		
Opening envelop with a number that corresponds to the results of the random group allocation	*X*		
Participants get allocated to a group	*X*		
Schedule a Visit	*x*		
Intervention		*x*	
Welcome by team assistant at the hospital entrance		*x*	
VRI set up and start the provision of service		*x*	
Demographics		*x*	
Medical History		*x*	
Physical Examination		*x*	
Measure height and weight		*x*	
blood pressure		*x*	
Application of the Communication scale performed by trained research team		*x*	
Assessmentss			*x*
Outcomes of health indicators assessments			*x*
Outcomes of Communication scale			*x*

ETHICAL AND REGULATORY ASPECTS

The current standard of interpretation provision is not efficacious or not acceptable to the patient.

In-person or VRI sign language interpretation is largely unavailable. There is no clear data on the availability of VRI or in-person interpretation. Given the cost, VRI may be more available than in person. Available data tend to focus on assessing personal references of Deaf users in regards to interpretation, as well as interpreters' preferences and maximising recourses allocation.

Three studies from the USA provide empirical data comparing VRI/ in-person interpretation

- One study found *no statistical difference* in their preference comparing VRI/ in-person interpretation for critical care but a statistical difference for non-critical care. - 103 participants in Chicago, Mixed methods- eight qualitative interviews
- A second study- An online survey exploring preferences- sample 189- In North Carolina found that respondents emphasised their preference for on-site interpreters, explaining how video remote interpreting was subject to technical difficulties.
- A secondary analysis of a national survey found that most people did not have access to VRI in healthcare settings. Almost half of the participants with access to VRI found the service satisfactory and the others unsatisfactory. Their conclusion claims that a fully functioning VRI system with qualified interpreters, this system can potentially reduce the number of emergency visits and unnecessary diagnostic tests, all of which are associated with cost burden.

Interpreters

A qualitative study found that interpreters preferred VRI because it allows them to allocate their time better. It enables interpretation agencies to maximise the use of specialised health interpreters

Patient Information and Informed Consent

The study will include only participants over 18 years old who communicate using sign language and hold enough sensorimotor, cognitive and communication skills to communicate independently with health personnel.

A formation sheet will be provided in written Spanish and a video in sign language and the consent form details the right to withdraw without providing an explanation, data security and privacy (see attached)

Participant privacy and confidentiality

Information shared during the consultation and the scale will be protected.

At first, information will be coded, and the researcher will a serial number and partial date of birth. After some tests have been run data will be fully anonymized the number that links the information back to you will be erased.

The results of this study may be published. Your name or any data that could be linked back to you will not be used in any publications that result from future scientific research.

Risks / Benefits

Serious adverse events

The trial is expected to be low risk for serious adverse events. While the risk is low, if there is

distress while in consultation occur, the PI (clinical team member onsite during all session times) and key Mederi staff will be immediately alerted, and the test will be immediately terminated if deemed necessary. There is a risk to find out about illness during the consultation, all participants are insured either by contributive or government-subsidence insurance schemes.

Benefits

Extra healthcare

Justification of choice of the study population

I have considered running a pre-post test of the intervention. A key limitation of this choice is that a not randomized study may not generalise well to the population. Given the availability of a participants list and infrastructure at partners hospital, an RCT addressing the following:

> ‘What is the effectiveness of the VRI system in improving communication outcomes between Deaf patients and doctors versus the ‘available standard of care of the usual communication tools, including informal interpretation, lip or note reading, using their mobile phones to contact a formal or informal interpreter, for Deaf patients aged 18 and older?

The randomization would allow unbiased assignment of participants to VRT and control group, therefore, making the study groups comparable. These results shall provide the needed evidence to address communication barriers experienced by Deaf persons while seeking healthcare. An RTC will provide not biased generalised evidence to address the gap of knowledge about the impact of the technology and to maximise resource allocation in health settings across LMIC.

Statistical methods

Participant demographics will be described both by group and overall sample. Responses for scale combined into two nominal categories (“Yes, probably Yes/ No, probably no) and differences between the intervention and control groups were analysed by Fisher’s exact test.

For secondary outcome measures, the main analysis will be comparing those indicators with non-Deaf persons using by Fisher’s exact test.
