# Supplementary material for: Video Remote Sign Language Interpreting and Health Communication for Deaf Patients: A Randomized Clinical Trial
Source: JAMA Netw Open. 2026 Feb 4;9(2):e2557189. doi: 10.1001/jamanetworkopen.2025.57189 (PMC12873765; doi:10.1001/jamanetworkopen.2025.57189)
Supplement: Supplement 2. — eTable. Ordinal Logistic Regression Fit Statistics and Mann-Whitney U Test (U Statistic and Median Scores) Results for 13 Communication Questions Comparing VRI Users and Nonusers [file jamanetwopen-e2557189-s002.pdf]

# Supplemental Online Content

Velarde MR, Izquierdo Martinez LC, Dalal J, et al. Video remote sign language interpreting and health communication for Deaf patients: a randomized clinical trial. *JAMA Netw Open.* 2026;9(2):e2557189. doi:10.1001/jamanetworkopen.2025.57189

**eTable.** Ordinal Logistic Regression Fit statistics and Mann-Whitney *U* Test (*U* Statistic and Median Scores) Results for 13 Communication Questions Comparing VRI Users and Nonusers

This supplemental material has been provided by the authors to give readers additional information about their work.

© 2026 Velarde MR et al. *JAMA Network Open*.

**eTable 1.** Ordinal Logistic Regression Fit statistics and Mann-Whitney *U* Test (*U* Statistic and Median Scores) Results for 13 Communication Questions Comparing VRI Users and Nonusers
AIC and log-likelihood indicate model fit; LRT p-values assess the proportional odds assumption. U-statistics and group medians summarize non-parametric comparisons.

| **Question** | **AIC** | **LogLik** | **LRT statistic** | **LRT (p-value)** | **U-statistic** | **Median (VRI)** | **Median (No VRI)** |
|---|---|---|---|---|---|---|---|
| Q1: Listening: | 427.29 | -209.65 | -127.70 | 1.00 | 5866.0 | 3.0 | 3.0 |
| Q2: Patiente | 468.67 | -230.34 | -254.63 | 1.00 | 5935.5 | 3.0 | 3.0 |
| Q3: Attentiveness | 494.14 | -243.07 | -228.28 | 1.00 | 6484.0 | 3.0 | 2.0 |
| Q4: Physical examination | 351.90 | -171.95 | -121.46 | 1.00 | 6350.5 | 3.0 | 3.0 |
| Q5: Empathy | 458.44 | -225.22 | -188.53 | 1.00 | 6195.0 | 3.0 | 2.0 |
| Q6: Clarity | 475.47 | -233.73 | -188.12 | 1.00 | 6067.0 | 3.0 | 2.5 |
| Q7: Completeness | 483.99 | -238.00 | -180.11 | 1.00 | 6450.0 | 3.0 | 2.0 |
| Q8: Disadvantages | 431.10 | -211.55 | -212.66 | 1.00 | 6210.0 | 3.0 | 3.0 |

© 2026 Velarde MR et al. *JAMA Network Open*.

| | | | | | | | |
|---|---|---|---|---|---|---|---|
| Q9: Decisional involvement | 507.56 | -249.78 | -218.99 | 1.00 | 6912.0 | 3.0 | 2.0 |
| Q10: Reassurance | 387.05 | -189.53 | -131.67 | 1.00 | 5950.0 | 3.0 | 3.0 |
| Q11: Understanding | 494.78 | -243.39 | -203.03 | 1.00 | 5938.0 | 3.0 | 2.5 |
| Q12: Confidence | 500.42 | -246.21 | -191.50 | 1.00 | 6264.0 | 3.0 | 2.0 |
| Q13: Concerns | 427.29 | -209.65 | -127.70 | 1.00 | 5866.0 | 3.0 | 3.0 |

© 2026 Velarde MR et al. *JAMA Network Open*.
