# Supplementary material for: Video Remote Sign Language Interpreting and Health Communication for Deaf Patients: A Randomized Clinical Trial
Source: JAMA Netw Open. 2026 Feb 4;9(2):e2557189. doi: 10.1001/jamanetworkopen.2025.57189 (PMC12873765; doi:10.1001/jamanetworkopen.2025.57189)
Supplement: Supplement 3. — Data Sharing Statement [file jamanetwopen-e2557189-s003.pdf]

Data Sharing Statement

Velarde. Video Remote Sign Language Interpreting and Health Communication for Deaf Patients. *JAMA Netw Open*. Published February 04, 2026.
doi:10.1001/jamanetworkopen.2025.57189

Data
Additional Information: ClinicalTrials.gov NCT05966623
https://clinicaltrials.gov/study/NCT05966623
Data available: Yes
Data types: Deidentified participant data
How to access data: Data-based hosted by research partner Universidad del Rosario
https://research-data.urosario.edu.co/dataset.xhtml?persistentId=doi:10.34848/HE2BFW
When available: With publication

Supporting Documents
Document types: Informed consent form
How to access documents: https://research-data.urosario.edu.co/dataset.xhtml?persistentId=doi:10.34848/HE2BFW
When available: With publication

Additional Information
Who can access the data: Upon reasonable request, Data will be shared for non-profit research purposes.
Types of analyses: Equity and non-discrimination research, quality of care and Disability and Deaf studies.
Mechanisms of data availability: a signed data access agreement
Any additional restrictions: Any research that may harm Deaf persons or other persons with disabilities or contradict Human rights principles.
